# Phytochemical and pharmacological investigation of *Spiraea chamaedryfolia*: a contribution to the chemotaxonomy of *Spiraea* genus

**DOI:** 10.1186/s13104-017-3013-y

**Published:** 2017-12-21

**Authors:** Tivadar Kiss, Kristóf Bence Cank, Orsolya Orbán-Gyapai, Erika Liktor-Busa, Zoltán Péter Zomborszki, Santa Rutkovska, Irēna Pučka, Anikó Németh, Dezső Csupor

**Affiliations:** 1 Department of Pharmacognosy, University of Szeged, Eötvös u. 6, Szeged, 6720 Hungary; 2 Interdisciplinary Centre for Natural Products, University of Szeged, Eötvös u. 6, Szeged, 6720 Hungary; 3 Department of Chemistry and Geography, Daugavpils University, Parādes st. 1, Daugavpils, 5401 Latvia; 4 Botanical Garden, University of Szeged, Lövölde u. 42, Szeged, 6726 Hungary

**Keywords:** Phytochemistry, Alkaloids, *Spiraea*, Antibacterial, Xanthine-oxidase, Chemotaxonomy

## Abstract

**Objective:**

Diterpene alkaloids are secondary plant metabolites and chemotaxonomical markers with a strong biological activity. These compounds are characteristic for the Ranunculaceae family, while their occurrence in other taxa is rare. Several species of the *Spiraea* genus (Rosaceae) are examples of this rarity. Screening *Spiraea* species for alkaloid content is a chemotaxonomical approach to clarify the classification and phylogeny of the genus. Novel pharmacological findings make further investigations of *Spiraea* diterpene alkaloids promising.

**Results:**

Seven *Spiraea* species were screened for diterpene alkaloids. Phytochemical and pharmacological investigations were performed on *Spiraea chamaedryfolia*, the species found to contain diterpene alkaloids. Its alkaloid-rich fractions were found to exert a remarkable xanthine-oxidase inhibitory activity and a moderate antibacterial activity. The alkaloid distribution within the root was clarified by microscopic techniques.

**Electronic supplementary material:**

The online version of this article (10.1186/s13104-017-3013-y) contains supplementary material, which is available to authorized users.

## Introduction

Plant metabolism, driven by photosynthesis, provides a huge number and a wide variety of natural products. These compounds are of great importance for their beneficial biological activities in humans. The investigation for specific plant metabolites is also a useful tool for the clarification of taxonomical uncertainties.

Diterpene alkaloids are secondary metabolites belonging to pseudoalkaloids [1]. This group of molecules includes numerous compounds with diverse skeletons and substitution patterns. These compounds can be classified according to the number of carbon atoms in the skeleton as bisnor-(C_18_), nor-(C_19_) and diterpene (C_20_) alkaloids. *Aconitum, Delphinium* and *Consolida* genera (Ranunculaceae) are known to be characterized by the presence of diterpene alkaloids. Although such alkaloids have also been reported from some *Inula* (Asteraceae), *Garrya* (Garryaceae), *Erythrophleum* (Fabaceae) and *Spiraea* (Rosaceae) species [2, 3], the occurrence of diterpene alkaloids in these taxa is sporadic. Since diterpene alkaloids are considered as chemotaxonomic markers [4], their presence in species other than those belonging to the Ranunculaceae family might have an important role in plant taxonomy.

The *Spiraea* genus, comprising approximately 100 species, belongs to the Rosaceae family. Phytochemical contents of 28 *Spiraea* taxa have been extensively studied. Mono-, di-, sesqui- and triterpenes have been isolated besides flavonoids, lignans, neolignans and other phenylpropane derivatives. Interestingly, only 9 of the investigated taxa were found to contain diterpene alkaloids *(S. formosana* Hayata, *S. fritschiana* var. *parvifolia* Liou, *S. japonica* L.f., *S. japonica* var. *acuta* Yu, *S. japonica* var. *fortunei* (Planchon) Rehder, *S. japonica* var. *glabra* (Regel) Koidz, *S. japonica* var. *incisa* Yu, *S. japonica* var. *ovalifolia* Zuo, *S. japonica* var. *stellaris)*. All of the reported 65 diterpene alkaloids bear hetisine- and atisine-type C_20_ basic skeletons (Additional file 1: *Spiraea* diterpene alkaloids). Although only marginal ethnomedicinal use of *Spiraea* species has been documented in North-America and Asia, pharmacological studies have reported noteworthy activities of *Spiraea* extracts and isolated compounds [5].

The recent classification and clarification of *Spiraea* phylogeny is based mainly on molecular analyses [6–9]. The phytochemical analysis is also considered as a useful tool to support plant classification.

Phytochemical studies on *Spiraea* genus are promising, because of their possible utilization as source of pharmacons. On the other hand, screening of this genus for diterpene alkaloid content may contribute to the clarification of *Spiraea* phylogeny. These considerations motivated our research, aiming to improve the current phytochemical knowledge on *Spiraea* species.

## Main text

### Materials and methods

#### Plant material

Seven *Spiraea* species were analysed. *S. crenata* L. (SZTE-FG 850) and *S. salicifolia* L. (SZTE-FG 851) were collected and identified by Gusztáv Jakab (Szent István University, Budapest, Hungary) in Hungary (Sepsibükszád and Alsórákos, Hungary). *S. nipponica* Maxim (SZTE-FG 852), *S.* x *vanhouttei* (Briot) Zabel (SZTE-FG 853) and *S.* x *billardii* hort. ex K. Koch (SZTE-FG 854) were collected and identified by Anikó Németh (Botanical Garden of University of Szeged, Szeged, Hungary). *S. media* Schmidt. (DAU 0 31 147 009) and *Spiraea chamaedryfolia* L. (DAU 0 31 145 023) were harvested in Daugavpils (Latvia), and identification was performed by Santa Rutkovska (University of Daugavpils, Latvia). Voucher specimens were deposited at the herbarium of the Department of Pharmacognosy of the University of Szeged and at that of the University of Daugavpils. Herb and root of the plant material were separated, dried and stored at room temperature until processing.

#### Extraction and identification of the alkaloid content

**Fig. 1:**
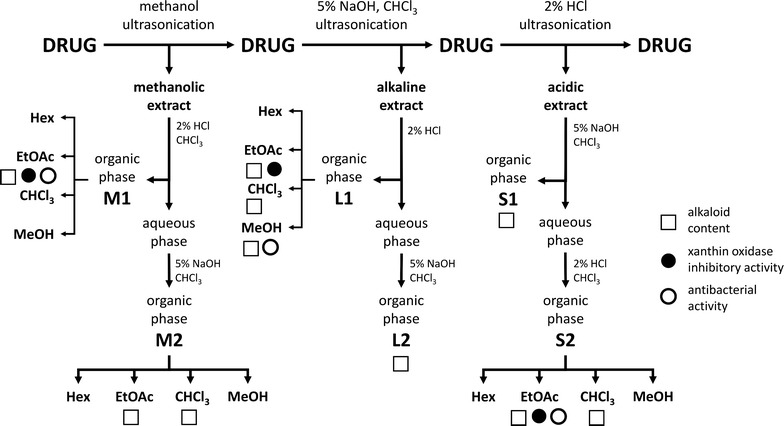
Alkaloid contents and pharmacological activities of *S. chamaedryfolia* fractions

Dried and crushed herb materials were extracted consequently with methanol (MeOH), chloroform (CHCl_3_) and 2% aqueous HCl, by ultrasonication at room temperature (Fig. 1). The applied drug-solvent ratio was 1:5 in each case. The drug was dried before each extraction phase. Moistening with 5% aqueous NaOH solvent was applied prior to extraction with chloroform.

The methanol extract was acidified with 2% aqueous HCl and was then extracted with chloroform. Fraction M1 was obtained by collecting and evaporating the organic phase. The pH of the aqueous phase was rendered to alkaline (pH 12) with 5% aqueous NaOH and was then extracted with chloroform. The chloroform phase yielded fraction M2.

The chloroform extract was further extracted with 2% aqueous HCl. The organic phase was evaporated and used as fraction L1. The pH of the aqueous phase was made alkaline and extracted with chloroform. The organic phase was evaporated to yield fraction L2.

The acidic extract was subjected to solvent–solvent partitioning with chloroform, after adjusting the pH to alkaline. The dry residue of the organic phase was labelled as S1. The pH of the aqueous phase was rendered to acidic with 2% aqueous HCl and was then extracted with chloroform. The organic phase was evaporated to produce fraction S2.

Fractions were screened for alkaloid content by thin layer chromatography (TLC), carried out at room temperature on silica gel (SiO_2_ 60 F254, Merck 1.05554.0001) and toluene/acetone/ethanol/*cc.*NH_3_ 70:50:18:4.5 was applied as mobile phase. Detection was performed in two steps: (1) dry plates were sprayed with Dragendorff’s reagent; and (2) after drying, the plates were sprayed again with 5% aqueous NaNO_2_. The alkaloids appeared as permanent brown spots.

#### Screening for antibacterial activity

Plant extracts were tested for antibacterial activity using the following microorganisms as test strains in the screening assays: 3 different Gram-positive strains, namely *Bacillus subtilis* (ATCC 6633), *Staphylococcus aureus* (ATCC 29213), and *Streptococcus pneumoniae* (ATCC 49619) plus one Gram-negative strain, namely *Moraxella catarrhalis* (ATCC 25238). In addition, the multi-resistant strain, methicillin-resistant *S. aureus* (MRSA, ATCC 43300) was used to test whether the fractions have a specific antibacterial effect on a strain of high public health priority. The test organisms were cultured on standard Mueller–Hinton agar plates or Columbia agar + 5% sheep blood (COS) plates (bioMérieux) at 37 °C. The bacterial cultures were maintained in their appropriate plates at 4 °C throughout the experiment and were used as stock cultures.

Antibacterial activities of our plant extracts were evaluated by the disc-diffusion method. The bacterial isolates for screening assay were prepared by picking single colony from 24 h old plates and it was suspended in sterile, isotonic saline solution (5 mL) to reach 0.5 McFarland standard of optical turbidity, resulting in a suspension containing approximately 1–2 × 10^8^ CFU/mL. The bacterial suspension was spread on appropriate sterile plates using a sterile cotton swab. Sterile filter paper discs (6 mm of diameter) were loaded with the extracts, using 20 μL of dried extracts redissolved in a mixture of ethanol and water (40/60 v/v) at a concentration of 50 mg/mL. After drying, these loaded filter paper discs were placed on the plates containing the bacterial suspensions. Paper discs impregnated with 20 µL of pure solvent were used as a negative control. The plates were then incubated at 37 °C for 24 h under aerobic conditions. Diameters of the inhibition zones produced by the plant extracts were measured and recorded (as the diameter of the inhibition zone plus the diameter of the disc) at 24 h.

#### Xanthine oxidase assay

The method is based on a continuous spectrophotometric rate determination: the absorbance of xanthine oxidase (XO) enzyme induced uric acid production from xanthine was measured at 290 nm for 3 min. The enzyme-inhibitory effect of our plant extracts was determined on the basis of the decrease in uric acid production. Reagents used included: 50 mM potassium buffer, pH 7.5 with 1 M KOH, 0.15 mM xanthine solution, pH 7.5, prepared using xanthine, XO enzyme solution 0.2 Units/mL prepared using XO. The test solutions applied included: *S. chamaedryfolia* fractions 12 g/mL, 600 µg/mL diluted in DMSO solution. The final reaction mixture of 300 µL well contained: 100 µL xanthine, 150 µL buffer and 50 µL XO for enzyme-activity. Allopurinol was dissolved in DMSO and used as positive control (100% inhibition was considered at 10 μg/mL concentration of allopurinol). The reaction mixture for inhibition: 100 µL xanthine, 140 µL buffer, 10 µL test and 50 µL XO.

#### Microscopical analysis

Specimens of the plant material were softened by ultrasonication in hot water for 1 h. Unembedded material was sectioned on a sledge microtome producing sections of 100 μm thickness. Observations were carried out on unstained sections. For histological characterisation 1% aqueous toluidine blue was used, and Dragendorff’s reagent was applied for alkaloid localisation. Transverse sections were mounted with water/glycerol 1:1. The sections were observed under light microscope and photographic images were captured using a digital camera.

### Results

Phytochemical screening revealed alkaloid content in *S. chamaedryfolia* roots, while all the other six *Spiraea* species were alkaloid-free. The solvent–solvent partitioning of methanolic, acidic and alkaline extracts of *S. chamaedryfolia* yielded alkaloid-rich ethyl acetate (EtOAc), chloroform and methanol fractions (Fig. 1). The most apolar fraction prepared with *n*-hexane (hex) was alkaloid-free. The attempt to isolate diterpene alkaloids have failed due to the low stability of the compounds.

The fractions were screened for in vitro antibacterial and xanthine oxidase inhibitory activity. The ethyl acetate fraction was found to be the most potent xanthine oxidase inhibitor, exerting over 70% of inhibition compared to allopurinol (Fig. 1 and Table 1).

**Table 1:** Antibacterial and xanthine oxidase inhibitory activities of *S. chamaedryfolia* fractions

Fractions	*Bacillus subtilis*	*Staphylococcus aureus*	*Streptococcus pneumoniae*	*Moraxella catarrhalis*	*Staphylococcus aureus* MRSA	*XO inhibition %*
M1-EtOAc	●	●	●	●	●	●
L1-MeOH	○	○	○	●	○	○
L1-EtOAc	○	○	○	○	○	●
S2-EtOAc	●	●	●	●	●	●

Fractions with activity (●) and fractions with no activity (○). (*EtOAc* ethyl acetate, *MeOH* methanol)

Three fractions were found to exert antibacterial activity against *S. aureus* (ATCC 29213), *B. subtilis* (ATCC 6633), *S. pneumoniae* (ATCC 49619), and *M. catarrhalis* (ATCC 25238), while one fraction exerted antibacterial activity against methicillin-resistant *S. aureus* (MRSA) (ATCC 43300) (Fig. 1 and Table 1).

**Fig. 2:**
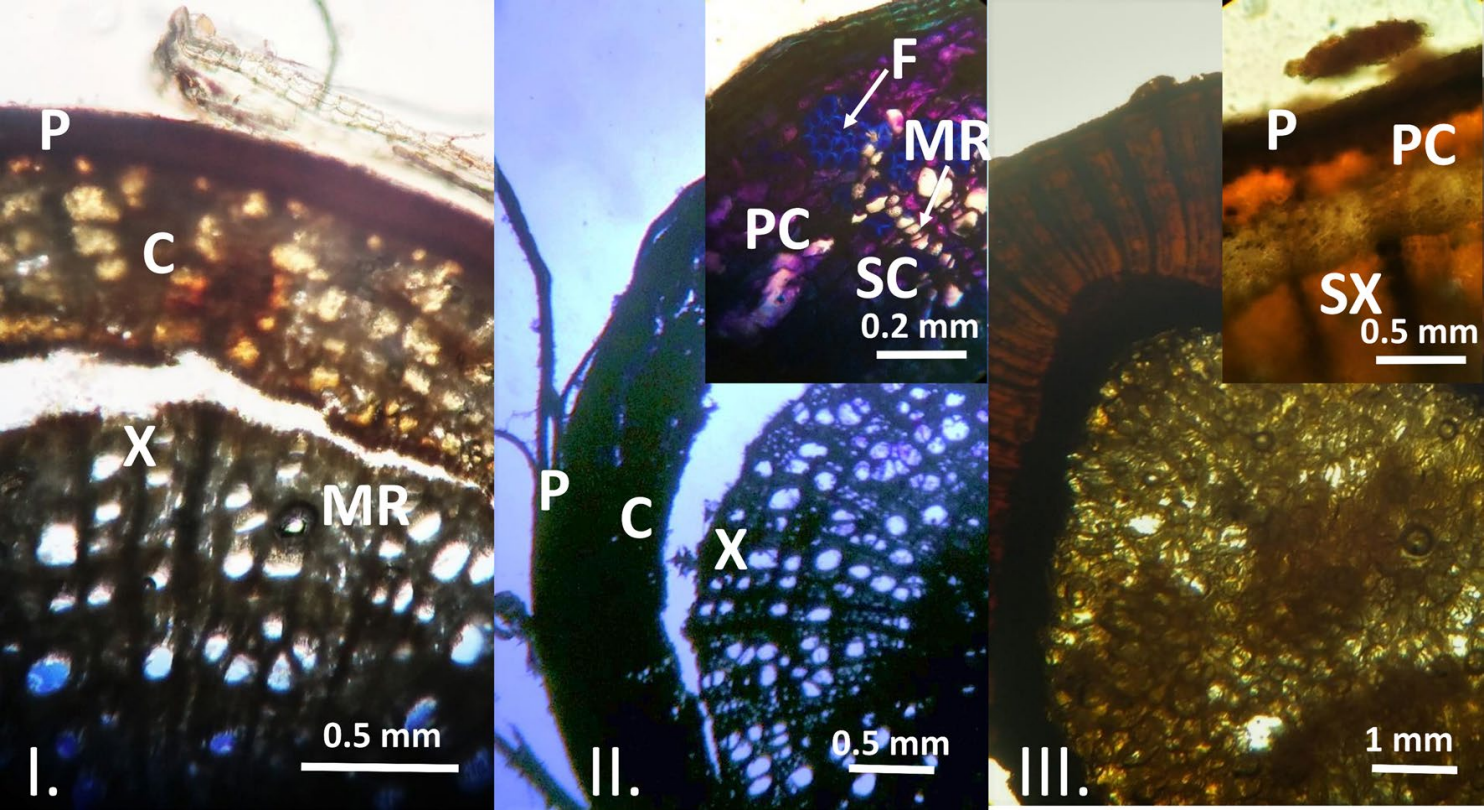
Transverse section of the root of *S. chamaedryfolia.* Transverse sections of the secondary root of *Spiraea chamaedryfolia,* unstained (I), stained with 1% toluidine blue (II) and treated with Dragendorff reagent (III). (*P* periderm, *C* cortex, *PC* primary cortex, *SC* secondary cortex, *X* xylem, *SX* secondary xylem, *MR* medullary ray)

Examining the transverse section of the root of *S. chamaedryfolia*, structures characteristic of secondary root were observed (Fig. 2). The periderm, primary and secondary cortex, and xylems with medullary rays could be observed in the unstained sections. Primary and secondary cortex with fibers in the primary cortex became visible after staining with toluidine blue. Dragendorff’s reagent revealed the presence of alkaloids in the secondary cortex and secondary xylem, while in the pith no signs of alkaloid content was observed.

### Discussion

Plants may contain alkaloids in two forms: either as free base or as salts of organic acids. The compounds present in the free base form can be extracted with organic solvents, while those in the salt form can be extracted using diluted inorganic acids. Diterpene alkaloids, and especially esters, may be unstable, thus they require special handling. For this reason alcoholic extraction is considered to be the most cautious method. However, the diverse structure and the substitution pattern of diterpene alkaloid molecules might require acidic and alkaline extraction as well. According to the literature, only alcoholic extraction was applied in previous phytochemical screening studies of *Spiraea* species, which might have resulted in an incomplete extraction. To prevent the decomposition of the alkaloid content, the order of extraction was determined to be started by methanol, and followed by organic and acidic extraction steps. The application of all these three extraction methods yielded fractions with a diverse alkaloid profile.

Unfortunately, although 4.0 kg of dried roots was used for the preparative phytochemical work, our efforts to isolate pure alkaloids were unsuccessful. After purification with adsorption chromatography (i.e. column chromatography and centrifugal planar chromatography) and gel filtration chromatography, the polarity and the molecular size of alkaloids and matrix compounds were similar within the obtained fractions, rendering separation impossible. Beside the notable amount of matrix compounds the highly unstable manner of alkaloids was also an obstacle to isolate pure compounds.

Fractions of *S. chamaedryfolia* were found to exert noteworthy biological activities. Xanthine oxidase inhibitory activity of *S. chamaedryfolia* fractions was remarkable, and the fractions also exerted a moderate antibacterial activity.

Proving the presence of alkaloids in *S. chamaedryfolia* is noteworthy, since only few taxa are known to have the ability to produce diterpene alkaloids: it has previously been reported for *S. japonica* 64 [10–29], *S. fritchiana* 2 [12, 16], *S. koreana* [30] and *S. formosa* 1 [31] only. No other types of alkaloids have been reported for the *Spiraea* genus. The alkaloid content of *S. chamaedryfolia* and the lack of alkaloids for *S. crenata, S. media, S. salicifolia, S. nipponica, S. x vanhouttei* and *S.* x *billardii* is first reported by our research group, making our phytochemical analyses pioneering in this field.

## Limitations

Only TLC detection methods were applied to confirm the alkaloid content, the subtypes of these alkaloids was not elucidated by LC–MS or NMR techniques. However, since no other alkaloid types have been reported from the *Spiraea* genus, this finding suggests the presence (or absence) of diterpene alkaloids in the investigated species.

## Additional file

**Additional file 1.** *Spiraea* diterpene alkaloids. Diterpene alkaloids reported from *Spiraea* genus.

## Abbreviations

C: cortex
CHCl_3_: chloroform
EtOAc: ethyl acetate
hex: hexane
LC–MS: liquid chromatography–mass spectroscopy
MeOH: methanol
MR: medullary ray
NMR: nuclear magnetic resonance
P: periderm
PC: primary cortex
SC: secondary cortex
SX: secondary xylem
TLC: thin layer chromatography
X: xylem
XO: xanthine oxidase
