# Supplementary material for: Phytochemical and pharmacological investigation of Spiraea chamaedryfolia: a contribution to the chemotaxonomy of Spiraea genus
Source: BMC Res Notes. 2017 Dec 21;10:762. doi: 10.1186/s13104-017-3013-y (PMC5740960; doi:10.1186/s13104-017-3013-y)
Supplement: Supplementary file 1 — Additional file 1. Spiraea diterpene alkaloids. Diterpene alkaloids reported from Spiraea genus. [file 13104_2017_3013_MOESM1_ESM.pptx]

## Slide 1
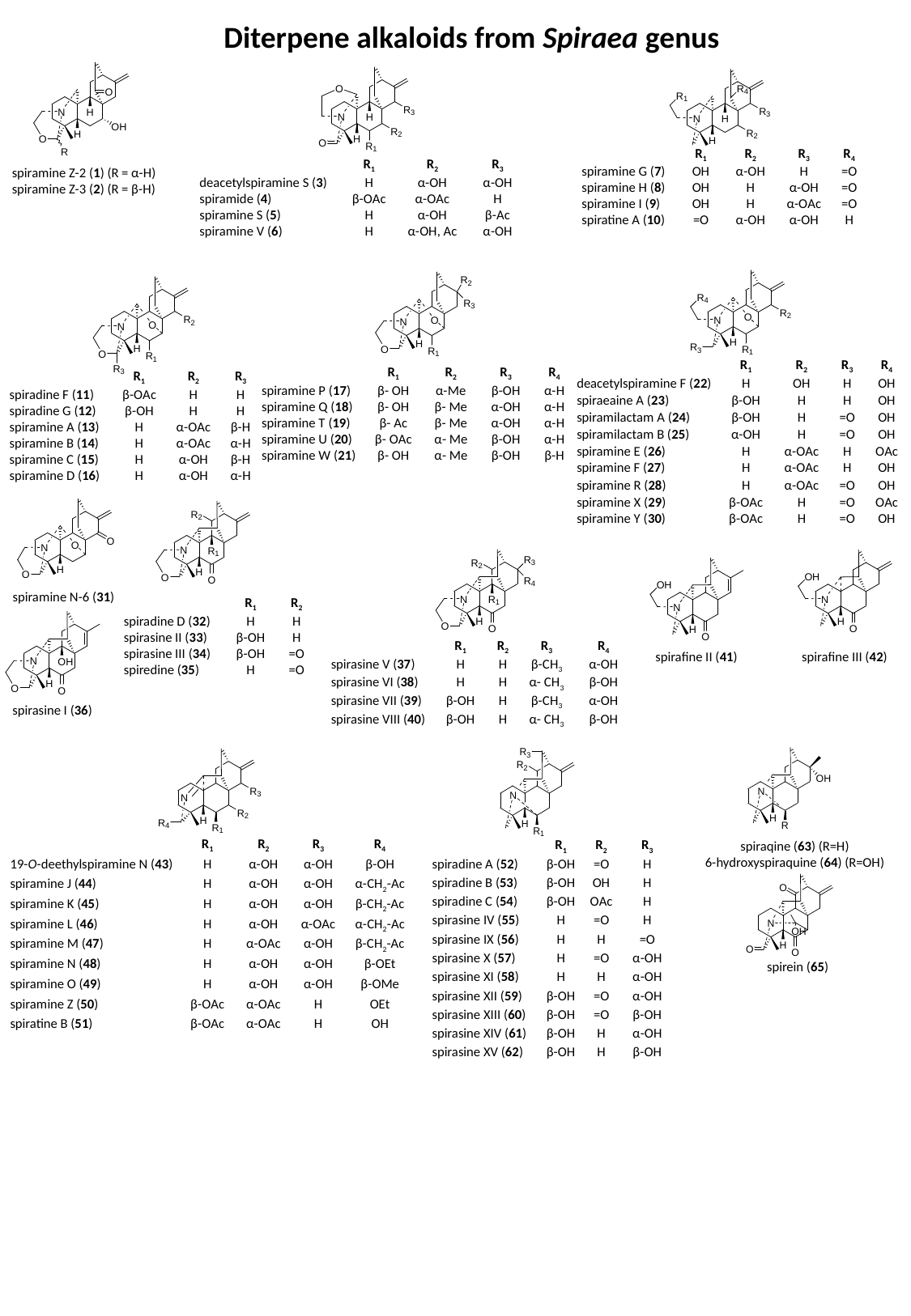

Diterpene alkaloids from Spiraea genus
| | R1 | R2 | R3 | R4 |
| --- | --- | --- | --- | --- |
| spiramine G (7) | OH | α-OH | H | =O |
| spiramine H (8) | OH | H | α-OH | =O |
| spiramine I (9) | OH | H | α-OAc | =O |
| spiratine A (10) | =O | α-OH | α-OH | H |
spiramine Z-2 (1) (R = α-H)
spiramine Z-3 (2) (R = β-H)
| | R1 | R2 | R3 |
| --- | --- | --- | --- |
| deacetylspiramine S (3) | H | α-OH | α-OH |
| spiramide (4) | β-OAc | α-OAc | H |
| spiramine S (5) | H | α-OH | β-Ac |
| spiramine V (6) | H | α-OH, Ac | α-OH |
| | R1 | R2 | R3 | R4 |
| --- | --- | --- | --- | --- |
| deacetylspiramine F (22) | H | OH | H | OH |
| spiraeaine A (23) | β-OH | H | H | OH |
| spiramilactam A (24) | β-OH | H | =O | OH |
| spiramilactam B (25) | α-OH | H | =O | OH |
| spiramine E (26) | H | α-OAc | H | OAc |
| spiramine F (27) | H | α-OAc | H | OH |
| spiramine R (28) | H | α-OAc | =O | OH |
| spiramine X (29) | β-OAc | H | =O | OAc |
| spiramine Y (30) | β-OAc | H | =O | OH |
| | R1 | R2 | R3 | R4 |
| --- | --- | --- | --- | --- |
| spiramine P (17) | β- OH | α-Me | β-OH | α-H |
| spiramine Q (18) | β- OH | β- Me | α-OH | α-H |
| spiramine T (19) | β- Ac | β- Me | α-OH | α-H |
| spiramine U (20) | β- OAc | α- Me | β-OH | α-H |
| spiramine W (21) | β- OH | α- Me | β-OH | β-H |
| | R1 | R2 | R3 |
| --- | --- | --- | --- |
| spiradine F (11) | β-OAc | H | H |
| spiradine G (12) | β-OH | H | H |
| spiramine A (13) | H | α-OAc | β-H |
| spiramine B (14) | H | α-OAc | α-H |
| spiramine C (15) | H | α-OH | β-H |
| spiramine D (16) | H | α-OH | α-H |
spiramine N-6 (31)
| | R1 | R2 |
| --- | --- | --- |
| spiradine D (32) | H | H |
| spirasine II (33) | β-OH | H |
| spirasine III (34) | β-OH | =O |
| spiredine (35) | H | =O |
| | R1 | R2 | R3 | R4 |
| --- | --- | --- | --- | --- |
| spirasine V (37) | H | H | β-CH3 | α-OH |
| spirasine VI (38) | H | H | α- CH3 | β-OH |
| spirasine VII (39) | β-OH | H | β-CH3 | α-OH |
| spirasine VIII (40) | β-OH | H | α- CH3 | β-OH |
spirafine II (41)
spirafine III (42)
spirasine I (36)
spiraqine (63) (R=H)
6-hydroxyspiraquine (64) (R=OH)
| | R1 | R2 | R3 | R4 |
| --- | --- | --- | --- | --- |
| 19-O-deethylspiramine N (43) | H | α-OH | α-OH | β-OH |
| spiramine J (44) | H | α-OH | α-OH | α-CH2-Ac |
| spiramine K (45) | H | α-OH | α-OH | β-CH2-Ac |
| spiramine L (46) | H | α-OH | α-OAc | α-CH2-Ac |
| spiramine M (47) | H | α-OAc | α-OH | β-CH2-Ac |
| spiramine N (48) | H | α-OH | α-OH | β-OEt |
| spiramine O (49) | H | α-OH | α-OH | β-OMe |
| spiramine Z (50) | β-OAc | α-OAc | H | OEt |
| spiratine B (51) | β-OAc | α-OAc | H | OH |
| | R1 | R2 | R3 |
| --- | --- | --- | --- |
| spiradine A (52) | β-OH | =O | H |
| spiradine B (53) | β-OH | OH | H |
| spiradine C (54) | β-OH | OAc | H |
| spirasine IV (55) | H | =O | H |
| spirasine IX (56) | H | H | =O |
| spirasine X (57) | H | =O | α-OH |
| spirasine XI (58) | H | H | α-OH |
| spirasine XII (59) | β-OH | =O | α-OH |
| spirasine XIII (60) | β-OH | =O | β-OH |
| spirasine XIV (61) | β-OH | H | α-OH |
| spirasine XV (62) | β-OH | H | β-OH |
spirein (65)
